# Moving the Fine Print to the Front Page: Transparent Communication of Facial Genetics Research

**DOI:** 10.1002/ggn2.202500056

**Published:** 2025-12-19

**Authors:** Jennifer K. Wagner, Nina Claessens, Caitlin M. Maloney, Peter Claes

**Affiliations:** ^1^ School of Engineering Design and Innovation Penn State University University Park PA USA; ^2^ Department of Electrical Engineering ESAT/PSI KU Leuven Leuven Belgium; ^3^ Medical Imaging Research Center University Hospitals Leuven Leuven Belgium; ^4^ Department of Anthropology Penn State University University Park PA USA; ^5^ Department of Human Genetics KU Leuven Leuven Belgium

**Keywords:** benchmarking, bioethics, DNA phenotyping, forensic science, policy

## Abstract

AI‐enabled facial genetics research has transformative potential for biomedical and forensic applications, but raises serious ethical, legal, and social challenges. Candor and clarity promote the advancement of science and facilitate the development of evidence‐informed and ethically sound policy guardrails for scientific applications. Expanding upon our recent Comment on Difface, here we examine some of the ethical dimensions of AI‐enabled facial genetics research, illuminate a lack of practical guidance for AI‐enabled facial genetics research, and contextualize potential impacts within the current legal and policy landscape. Given the lack of standards and benchmarks for DNA‐based face generation, we use Difface as a case study to stress the need for transparent performance metrics and clear disclosure of data flows across AI training, validation, and testing pipelines—enabling non‐experts to assess accuracy meaningfully. Finally, we offer practical guidance for scientists to promote trustworthiness and stimulate further discussion within professional societies—guidance urgently needed in sociopolitically‐turbulent and deregulatory environments. Risks of misinformation, disinformation, and discriminatory application of facial genetics research are too serious to ignore.

## Graphical abstract

This Perspective uses the recent Difface model for DNA facial phenotyping as a springboard to critically examine some of the Ethical, Legal, and Social Implications (ELSI) of AI‐enabled Facial Genetics research. It illuminates a Lack of Practical Guidance Offered By Relevant Professional Societies and Underscores the Importance of Concurrent Handling of Technical and ELSI matters. A discussion Draft ELSI Checklist for AI‐enabled Facial Genetics Research is offered, inviting a broader conversation about transparency, benchmarking, and responsible communication, urging professional societies to move such considerations from the fine print to the front page.

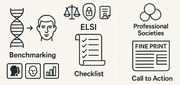

## 1 Introduction

Artificial intelligence (AI) and AI‐enabled science and technology have been accelerating rapidly in recent years. Among them are AI‐enabled facial genetics research and facial recognition technologies, which offer considerable potential for a wide range of applications (including law enforcement, national security, immigration, humanitarian, healthcare, and anthropological research, amongst others). Corresponding public policy around the world for the responsible development and use of AI‐enabled technologies has been slowly but steadily emerging as well [1]. The most notable development, perhaps, has been the emergence of the EU AI Act in Europe. AI laws have now also come to fruition in South Korea [2] and Japan [3, 4], and there is a flurry of legislation under intense debate around the world [1]. While no federal AI laws have yet been enacted in the United States, emerging AI policy (see Box 1) has included, for example, the 2022 Blueprint for an AI Bill of Rights issued by the White House Office of Science and Technology Policy [5], the 2023 Executive Order on Safe, Secure, and Trustworthy Development and Use of Artificial Intelligence issued by President Biden [6], the subsequent 2024 Office of Management and Budget's Memoranda implementing that executive order and directing governmental agencies on the use of AI, a variety of relevant documents from the Department of Health and Human Services generally and Food and Drug Administration [7] specifically, Federal Trade Commission guidance, reports, and actions, several state AI laws [8], and a host of normative guidance documents from expert advisory bodies (including the National Academy of Medicine). This approach to develop and implement reasonable guardrails for the wide array of applications for AI‐enabled science and technology has encountered an abrupt U‐turn in the United States, with a slew of Executive Orders issued in 2025 by President Trump (shown in Box 2) [9, 10, 11, 12, 13, 14, 15, 16, 17, 18, 19, 20] and legislative proposals [21, 22] to impose moratoria on state or local AI governance, signaling that we have entered a volatile and extreme deregulatory era. Furthermore, anticipated governmental actions in the United States to accelerate AI innovation—e.g., to establish “a dynamic, ‘try‐first’ culture for AI” (including in “critical sectors” such as healthcare), to invest in the science of AI as well as AI‐enabled science, and to create “the world's largest and highest quality AI‐ready scientific datasets…” as called for in America's AI Action Plan [23] issued in July 2025—could lead to considerable confusion and legal uncertainty.

**BOX 1 | Highlights of emerging AI policy in the United States**

**Table:** 

State Laws	Office of Management and Budget	Federal Trade Commission	Department of Health and Human Services	Food and Drug Administration	National Academy of Medicine
– Utah [24] – Colorado [25] – California [26] – Texas [27]	*Memoranda*: “Advancing Governance, Innovation, and Risk Management for Agency Use of Artificial Intelligence” [28]	*Guidance*: – “Keep your AI claims in check” [29] – “AI companies: uphold your privacy and confidentiality commitments” [30] – “AI and the Risk of Consumer Harm” [31] *Reports*: – “FTC Staff Report on AI Partnerships & Investments 6(b) Study” [32] *Actions*: – “Operation AI Comply” [33]	– AI Strategic Plan [34] *Office of Civil Rights clarifications*: – Final Rule, Section 1557 Affordable Care Act [35] – Dear Colleague Letter “Re: Ensuring Nondiscrimination Through the Use of Artificial Intelligence and Other Emerging Technologies” [36]	– SaMD Discussion Paper [37] – SaMD Action Plan [38] – Good Machine Learning Practice for Medical Device Development: Guiding Principles [39] – Clinical Decision Support Software Guidance [40] – Draft Guidance: Predetermined Change Control Plan (PCCP) [41] – Drug Development Discussion Paper [42] – PCCP Guiding Principles [43] – Artificial Intelligence and Medical Products: How CBER, CDER, CDRH, and OCP are Working Together Report [44] – Transparency for Machine Learning‐Enabled Medical Devices: Guiding Principles [45] – PCCP Final Guidance [46] – Lifecycle Management Draft Guidance [47] – Considerations for the Use of Artificial Intelligence to Support Regulatory Decision‐making for Drug and Biological Products [48]	– Discussion Draft AI Code of Conduct and Principles [49] – Finalized AI Code of Conduct and Principles [50]

**BOX 2 | Sampling of recent executive orders with possible implications for AI‐based facial genetics research and applications in the United States**

**Table:** 

*January 2025* Exec. Order No. 14148: “Initial Rescissions of Harmful Executive Orders and Actions” (issued 1/20/2025) Exec. Order No. 14151: “Ending Radical and Wasteful Governmental DEI Programs and Preferencing” (issued 1/20/2025) Exec. Order No. 14168, “Defending Women from Gender Ideology Extremism and Restoring Biological Truth to the Federal Government” (issued 1/20/2025) Exec. Order No. 14173: “Ending Illegal Discrimination and Restoring Merit‐Based Opportunity” (issued 1/21/2025) Exec. Order. No. 14179: “Removing Barriers to American Leadership of Artificial Intelligence” (issued 1/23/2025) Exec. Order. No. 14192: “Unleashing Prosperity Through Deregulation” (issued 1/31/2025) *March 2025* Exec. Order. No. 14243: “Stopping Waste, Fraud, and Abuse by Eliminating Information Silos” (issued 3/20/2025) *May 2025* Exec. Order No. 14303: “Restoring Gold Standard Science” (issued 5/23/2025) *July 2025* Exec. Order No. 14318: “Accelerating Federal Permitting of Data Center Infrastructure” (issued 7/23/2025) Exec. Order No. 14319: “Preventing Woke AI in the Federal Government” (issued 7/23/2025) Exec. Order No. 14320: “Promoting the Export of the American AI Technology Stack” (issued 7/23/2025) *August 2025* Exec. Order No. 14332: “Improving Oversight of Federal Grantmaking” (issued 8/7/2025)

Of the >200 executive orders issued by the Trump administration between January 20, 2025, and October 15, 2025, on a wide array of issues, this box displays the dozen with direct implications for AI‐related science and technology research, development, and applications. Executive orders are binding directives on federal government agencies (such as the FDA, FTC, DHHS, DOJ, FBI, etc.) that require agency policies, programs, and practices to be in alignment.

Accordingly, stable and robust public policy for AI governance generally–i.e., government‐mandated rights and duties–is unlikely for the foreseeable future. Even if legislative or regulatory action were to occur, deviations and abandonment from long‐standing precedents indicate the judiciary is increasingly scrutinizing administrative decisions and limiting agency authority and interpretive deference [59, 60]. There are shifting, unclear, and uncertain expectations of AI‐enabled facial genetics for health purposes. Similarly, there are stagnant, eroding, or uncertain expectations for AI‐enabled facial genetics for forensic purposes (with persistent calls for any forensic science to be both more robust [61, 62] and open [63, 64] and with varying legal and regulatory constraints on forensic DNA phenotyping around the world [57, 58, 65] as summarized in Table 1). Technical debts and moral debts are compounding, and time is of the essence to address a wide variety of challenges (e.g., problems posed by exceptionalism of genetic, neural, or other types of information, the need for equitable and uniform consent processes, proxy discrimination and disparate impacts of AI, functional obsolescence of HIPAA [66] and its Privacy Rule exceptions, unreasonable expectations on data privacy given the HIPAA knowledge gap, the illusion of “de‐identification” given our datafied cultures and technological capabilities, dual use challenges, scant normative constraints on DNA phenotyping, etc.). The anticipated increase in litigation and proliferation of both legislative impasses and regulatory ambiguities discourage investment. The legal gaps and uncertainties impede scientific and technological innovation [67].

**TABLE 1:** A Glimpse of the legal landscape involving facial DNA phenotyping for law enforcement purposes.

Country	Legal status
United States	While not authorized for use or integration with CODIS DNA samples, there is no explicit federal law or policy to prohibit, permit, or otherwise regulate facial DNA phenotyping practices [51]. Some states (such as New York) have been trying to pass legislative bans on facial DNA phenotyping [52].
Netherlands	DNA phenotyping is explicitly permitted but only for traits for which parliamentary approval has been given (which has not yet expanded beyond eye, hair, and skin color to authorize phenotyping of facial feature shapes).
Germany	In 2019, the German Code on Criminal Procedure § 81e was amended to allow DNA phenotyping for specific externally visible traits (which appear to be limited to eye, hair, and skin color and information related to age) [53].
Switzerland	The Swiss DNA Profiles Act was revised in 2023 to allow DNA phenotyping of certain externally visible traits (which appears to be limited to eye, hair, and skin color and information related to age and DNA ancestry) and enable additional traits (such as facial feature shapes) to be added to the permissible list by the Swiss Federal Council without further parliamentary approval [54, 55].
France	There is no explicit legislation permitting DNA phenotyping and implicit regulation prohibiting DNA analysis beyond identification; however, an exception created in a legal case decided in 2014 allows for inference of “morphological characteristics” of suspects in criminal investigations. There has been legal ambiguity as to which traits are permissible through this exception [56, 57, 58].
United Kingdom, Poland, Czech Republic, Sweden, Hungary, Austria, Spain	These countries reportedly [57] have practiced forensic DNA phenotyping “in compliance with existing laws” as of December 2019.

All of this underscores the importance of robust and vigilant self‐governance strategies (relying less on statutory law and more on contractual obligations) to ensure the rigorous and responsible conduct of AI‐enabled facial genetics research as well as the responsible communication and application of AI‐enabled facial genetics science and technologies in society. Practice standards, guidelines, recommendations, codes of ethics, and codes of conduct on point issued by scientific professional societies and organizations have the power to impel desirable and deter undesirable behaviors and actions not otherwise required by law. Unfortunately, such normative guidance on AI‐enabled facial genetics research generally and facial DNA phenotyping specifically seems to be unavailable to date. For example, a search of published standards [68] by the American Academy of Forensic Sciences (AAFS) for “phenotyping” standards on June 28, 2025, revealed no results. A similar search for guidance by the International Society of Forensic Genetics (ISFG) uncovered no publicly accessible policy guidance despite posting forensic DNA phenotyping tools to the website for the past four years [69, 70]. A search of American College of Medical Genetics and Genomics (ACMG) practice resources [71] (specifically policy statements and practice guidelines) for the keyword “phenotyping” revealed a 2018 policy statement that unfortunately does not address facial features [72]. Literature searches performed in August 2025 specifically to uncover relevant normative guidance published in key pre‐selected academic journals (i.e., American Journal of Human Genetics, Human Genetics and Genomics Advances, Genetics in Medicine, European Journal of Human Genetics, and American Journal of Biological Anthropology) were unsuccessful. Stated more directly, we are unaware of any professional practice guidance directly on the subject of predicting facial appearance or externally visible traits issued by the American Society of Human Genetics (ASHG) [73, 74], the European Society of Human Genetics (ESHG), the American College of Medical Genetics and Genomics (ACMG), the American Association of Biological Anthropologists (AABA), the International Society of Forensic Genetics (ISFG) [75], the American Association of Forensic Sciences (AAFS), the International Association of Forensic Sciences (IAFS), or even the Scientific Working Group on DNA Analysis Methods (SWGDAM). This is a serious gap. Our work here is intended not to settle the issues once and for all but to spark interest, discussion, and policy development amongst the relevant professional societies, associations, and organizations.

Among our recognized international human rights are the human rights to health [76, 77], science [78, 79], and privacy [80, 81, 82, 83]. These need not be viewed in conflict with one another, nor do they exist in any inherent ranked order. Rather, the three are intertwined and must be advanced together, aligning our values to pursue the realization of all three in concert, such that we avoid hyposurveillance of the data poor and hypersurveillance of the data rich. Such challenges and opportunities are exemplified by AI‐enabled facial genetics research. The United States and other state parties to international human rights treaties have obligations to respect, protect, and fulfill these fundamental rights and have the benefit of elaborations on how the right to science specifically can be advanced [84]. Regardless of governmental action and inaction, it is incumbent upon researchers engaged in AI‐enabled facial genetics to anticipate the implications of their work not only in their fields and applications but other areas of society and take measures to mitigate potential misunderstandings and misapplications of that work. It seems appropriate, therefore, that various ethical dimensions of science (i.e., intrinsic, extrinsic, and procedural) [85] be examined and discussed concurrently when reporting AI‐enabled facial genetics research rather than relegated to separate scholarly spheres.

Research and real‐world applications of DNA‐based facial phenotyping have sparked intense criticism. In 2017, Craig Venter and colleagues at Human Longevity, Inc. faced backlash to their article on predictive genetic reidentification [86]; this included claims that the authors overstated model feasibility and the significance of the results, as well as authors’ conflicts of interest in the research outcomes and peer‐review process [87, 88]. Parabon NanoLabs has been at the center of several controversies in facial genetics research [89], notably in 2020 when police attempted to use facial recognition technology on a profile Parabon created using their DNA phenotyping technology, which, of concern, remains unvalidated to this day [90]. Similarly, we are concerned with the development and implications of Difface, an AI‐enabled facial reconstruction model published by Jiao et al. [91] earlier this year.

There is an understandable appeal and intense attraction to the possibility that AI‐enabled facial genetics research might one day be capable of confidently and reliably predicting facial appearance from DNA–not only for law enforcement purposes but also humanitarian purposes (e.g., disaster victim identification) and anthropological (e.g., paleontological, archaeological [92], and evolutionary) research purposes. In this appeal, however, considerable danger lurks. AI‐enabled facial genetics research must be thorough and transparent in disclosure and explanation of the capabilities and limitations of the methods, as well as the practical meaning of the results. In addition to qualitative evaluations (such as red teaming [93]), quantitative performance evaluations (i.e., benchmarking [94] and computational checklists [95, 96]) have become broadly recognized as playing an important role in assessing risks of harm from AI technologies and ensuring accuracy, fairness, and trustworthiness of AI. While benchmarking is not without its own critics and, moreover, while an over‐reliance on benchmarking results warrants their use only with caution [97], such tools offer valuable starting points for common understanding and deliberations.

Here, we offer practical guidance for scientists engaged in facial genetics research to validate and contextualize the performance of the systems developed.

## 2 Proposed Checklist for AI‐Enabled Facial Genetics Research

Adherence to established best practices in machine learning is not optional but necessary. This begins with the clear separation of training, validation, and test datasets, each serving a distinct role: training data to build the model, validation data to tune hyperparameters, and test data to assess generalizability to unseen cases. Within the context of facial genetics, the discovery cohort in a GWAS may be reasonably aligned with the training set, the replication cohort with the validation set, and a fully independent dataset should be reserved for final testing. The requirement for three independent datasets, however, exposes a critical limitation: most current datasets in facial genetics are insufficient in size to support this structure. As such, the development of larger, more diverse, and ethically sourced datasets is a necessary next step for advancing the field of AI‐enabled DNA facial phenotyping in a scientifically sound and socially responsible manner.

Looking ahead, and considering the analyses presented above, we strongly recommend the routine implementation and transparent reporting of benchmarked performance metrics, including error bounds and interpretable baseline models. Such models include, but are not limited to, those simulating random guessing to establish the typical range of facial differences between unrelated individuals, which sets a baseline for non‐informative prediction. Another, more challenging, benchmark is defined by the average facial difference between the most similar individuals in the dataset, representing the lowest achievable error when matching different people. For a prediction model to truly claim individual‐level accuracy, its reconstruction error should fall below this lower bound. In other words, the predicted face should resemble the true face more closely than any other face in the dataset does. Additionally, baseline models using straightforward machine learning techniques and demographic variables, such as sex, age, BMI, and background, help delineate the threshold below which performance is likely driven by demographic information augmented with genetic facial features.

These benchmarks have been systematically applied and illustrated in our commentary on the recent Difface model [98], offering a solid foundation for future studies. In brief, although Difface reported a reconstruction error of 3.52 mm for faces predicted from individual genetic markers, a figure that appears low in absolute terms and was widely emphasized in media coverage [99, 100], its meaning only becomes clear when evaluated against interpretable benchmarks. Specifically, this error was only marginally better than the average difference between randomly paired faces (benchmark 1: 3.66 mm), and substantially worse than the average difference between the most similar individuals in the dataset (benchmark 2: 1.63 mm). Moreover, a baseline model using only demographic variables and partial least squares regression (benchmark 3: 2.33 mm) outperformed Difface, suggesting that demographic similarity alone may account for much of the predictive signal. These findings indicate that Difface reconstructions currently lack the precision required for dependable individual‐level identification. Benchmarking practices such as these are essential not only for contextualizing performance claims but also for informing potential users and stakeholders about the practical value and limitations of DNA‐based facial reconstruction technologies.

Equally important is the comprehensive documentation of data usage across all stages of the research pipeline, from initial preprocessing to final evaluation. This level of transparency is critical for ensuring reproducibility, fostering trust, and enabling meaningful peer review. Moreover, the tone used to communicate findings, particularly in results and discussion sections, should be carefully calibrated to reflect the actual level of performance achieved, avoiding overstatement or premature claims of readiness.

That said, it is important to acknowledge and commend studies such as Jiao et al. that subject their systems to academic scrutiny through peer‐reviewed publication and open experimentation. This stands in stark contrast to proprietary or commercial systems that operate without such validation [101]. Based on the current state of evidence in facial genetics, it is reasonable to conclude that DNA‐based facial phenotyping remains a proof of concept. Its practical utility is, at present, unproven, and substantial methodological and ethical development is still required before real‐world deployment can be responsibly considered. As stated by Hallgrimsson et al. [102], in response to some of our own work [103], “Let's Face It, complex traits are just not that simple.”

**BOX 3 | Discussion draft checklist for AI‐enabled facial genetics research**

**Table:** 

Is terminology (e.g., about data and methods) clearly defined and consistently used? Is there a validated partitioning of the data for training, validation, and testing, and has this partitioning been explicitly illustrated? Have robust and replicable descriptions of features (such as SNP selection) been provided? Have acceptable performance metrics and their limitations of AI‐based approaches been clearly articulated and explained via comparison to a well‐recognized, easily interpretable baseline model (e.g., AI model vs. standard machine learning, e.g., partial least squares‐, LASSO‐, or principal component regression) Has the diversity and representativeness of the data been disclosed, and what steps have been taken to identify and mitigate the risk of bias? Have bad examples been presented along with good examples of the AI‐based approach's output and described performance? Bad examples include individual cases with the lowest reconstruction accuracy or recognition ability, as well as particular (under‐represented) groups with a systematic lower performance compared to other groups. Is it clearly indicated that the output is AI‐generated, and has this been consistently communicated across examples and figures? Have uncertainties in the predicted output been adequately conveyed, both in terms of model limitations and data variability? This could include visual cues such as blurred regions in facial reconstructions where prediction confidence is low or presenting multiple plausible outputs to reflect variability due to model parameters or incomplete input data. Providing only a single, unqualified output might give a misleading impression of accuracy or precision. Are the researchers supporting broad or open sharing of code and data? What are the possibilities for ambiguity, confusion, or intentional and unintentional misinterpretation of the data or algorithm, and what steps have been taken to identify and mitigate those risks?

In the absence of government‐imposed mandates or legal requirements, researchers often turn to professional practice guidance and industry‐generated standards to determine whether science and technology are robust and their application appropriate. Checklists–or “points to consider” documents, as some organizations are accustomed to issuing–could cover a wide range of important contextual considerations. We recognize that researchers are sometimes reluctant to address such issues (e.g., viewing the subject matter as outside of their area of expertise and for others to address). Other times, even conscientious researchers relegate such information to the “fine print” buried in annexed back matter (e.g., viewing the subject matter as tangential rather than mission‐critical or seeing no viable alternative when trying to comply with rigid journal constraints capping word counts and reference citations below what would be required for a holistic approach). We personally have found ourselves in this very situation—not only with our prior work, e.g. [103], but also our current attempt to offer constructive dialogue regarding Difface [98]. Nevertheless, we argue these considerations would better be addressed front and center. The preliminary checklist we offer in Box 3 is intended as a work‐in‐progress, shared for the sole purpose of inspiring spirited discussions about what elements or features, topics, and subtopics such tools should be documented and communicated. Checklist implementation also raises a host of issues to consider, including, e.g., determining an appropriate level of content coverage without overburdening researchers, identifying and mitigating risks that checklists would be “gamed,” and assessing whether such checklists have their intended effect to improve the quality and quantity of attention given to these matters.

As we noted previously, we do not expect this contribution to settle the challenging issues with any finality, whether within health or forensic spaces involving AI‐enabled facial genetics research. Indeed, we see value in crowd‐sourcing a dynamic checklist or ethical, legal, and social implications (ELSI) resources from experts and members across the many areas of AI‐enabled facial genetics and invite the many potential professional organizations that could–and we argue should–undertake efforts to impel responsible AI‐enabled facial genetics practices to take up this important cause.

## Funding

This work was supported in part by the Research Program of the Research Foundation‐Flanders (FWO, G0D1923N to PC).

## Conflicts of Interest

The authors declare no conflicts of interest.

## Supporting information




**Supporting File**: ggn270019‐sup‐0001‐SuppMat.docx.
