# Supplementary material for: Moving the Fine Print to the Front Page: Transparent Communication of Facial Genetics Research
Source: Adv Genet (Hoboken). 2025 Dec 19;6(4):e00056. doi: 10.1002/ggn2.202500056 (PMC12747527; doi:10.1002/ggn2.202500056)
Supplement: Supplementary file 1 — Supporting File: ggn270019‐sup‐0001‐SuppMat.docx. [file GGN2-6-e00056-s001.docx]

**Supporting Information 1. Methods for Locating Relevant Professional Practice Guidance**

To identify relevant professional practice guidance for AI-enabled DNA-based facial phenotyping, we conducted several online searches of websites for relevant professional societies and searched the archives of a pre-selected set of journals where such normative guidance would be expected if available.

In June 2025, one researcher (JKW) conducted searches directly on the websites of the following professional societies, organizations, and associations to identify any published practice standards, guidelines, recommendations, position statements, or policies on point:

1. American Academy of Forensic Sciences, <https://www.aafs.org/search/standards?_page=1&keywords=phenotyping&_limit=9&status_standard=4>
2. International Society of Forensic Genetics, <https://www.isfg.org/Phenotyping> and <https://www.isfg.org/News;419>
3. American College of Medical Genetics and Genomics, <https://www.acmg.net/ACMG/Medical-Genetics-Practice-Resources/Documents_in_Development.aspx>
4. American Society of Human Genetics, <https://www.ashg.org/tag/guidance/>
5. Scientific Working Group on DNA Analysis Methods, <https://www.swgdam.org/publications>

The searches were unsuccessful in locating any relevant documents.

In August 2025 to augment those searches, one researcher (CMM) explored the journal archives for any formally published practice standards, guidelines, recommendations, position statements, or policies on point specifically in five prominent journals:

1. American Journal of Human Genetics <https://www.cell.com/ajhg/home>
2. Human Genetics and Genomics Advances (an official journal of ASHG), <https://www.cell.com/hgg-advances/home>
3. Genetics in Medicine (an official journal of ACMG), <https://www.gimjournal.org/>, <https://www.sciencedirect.com/journal/genetics-in-medicine/issues>, and <https://www.sciencedirect.com/journal/genetics-in-medicine-open>
4. European Journal of Human Genetics (an official journal of the European Society of Human Genetics), <https://www.nature.com/ejhg/>
5. American Journal of Biological Anthropology (an official journal of the American Association of Biological Anthropologists), <https://onlinelibrary.wiley.com/journal/26927691>

Three distinct search strings were used: (1) “facial dna phenotyping”; (2) “facial phenotyping”; and (3) "facial" AND "DNA" AND "phenotyping”. For results from each search, titles were screened manually by CMM to identify potentially relevant results for closer review. While these searches returned a total of N=782 results, none (n=0) passed the initial screening of titles to warrant a more detailed inspection. See Table 1 below.

Table 1. Unsuccessful searches of five journals

| **Date of Search** | **Journal** | **Website** | **Search String Used** | **No. of results** | **No. of potentially relevant results** |
| --- | --- | --- | --- | --- | --- |
| 8/14/2025 | American Journal of Human Genetics | <https://www.cell.com/ajhg/home> | “facial dna phenotyping” | 0 | 0 |
| 8/14/2025 | American Journal of Human Genetics | <https://www.cell.com/ajhg/home> | “facial phenotyping” | 5 | 0 |
| 8/14/2025 | American Journal of Human Genetics | <https://www.cell.com/ajhg/home> | “facial” AND “DNA” AND “phenotyping” | 109 | 0 |
| 8/14/2025 | Human Genetics and Genomics Advances | <https://www.cell.com/hgg-advances/home> | “facial dna phenotyping” | 0 | 0 |
| 8/14/2025 | Human Genetics and Genomics Advances | <https://www.cell.com/hgg-advances/home> | “facial phenotyping” | 0 | 0 |
| 8/14/2025 | Human Genetics and Genomics Advances | <https://www.cell.com/hgg-advances/home> | “facial” AND “DNA” AND “phenotyping” | 10 | 0 |
| 8/14/2025 | Genetics in Medicine | <https://www.sciencedirect.com/journal/genetics-in-medicine/issues> | “facial dna phenotyping” | 0 | 0 |
| 8/14/2025 | Genetics in Medicine | <https://www.sciencedirect.com/journal/genetics-in-medicine/issues> | “facial phenotyping” | 26 | 0 |
| 8/14/2025 | Genetics in Medicine | <https://www.sciencedirect.com/journal/genetics-in-medicine/issues> | “facial” AND “DNA” AND “phenotyping” | 376 | 0 |
| 8/14/2025 | Genetics in Medicine | <https://www.gimjournal.org/> | “facial dna phenotyping” | 0 | 0 |
| 8/14/2025 | Genetics in Medicine | <https://www.gimjournal.org/> | “facial phenotyping” | 2 | 0 |
| 8/14/2025 | Genetics in Medicine | <https://www.gimjournal.org/> | “facial” AND “DNA” AND “phenotyping” | 84 | 0 |
| 8/14/2025 | Genetics in Medicine Open | <https://www.sciencedirect.com/journal/genetics-in-medicine-open> | “facial dna phenotyping” | 0 | 0 |
| 8/14/2025 | Genetics in Medicine Open | <https://www.sciencedirect.com/journal/genetics-in-medicine-open> | “facial phenotyping” | 7 | 0 |
| 8/14/2025 | Genetics in Medicine Open | <https://www.sciencedirect.com/journal/genetics-in-medicine-open> | “facial” AND “DNA” AND “phenotyping” | 48 | 0 |
| 8/14/2025 | European Journal of Human Genetics | <https://www.nature.com/ejhg/> | “facial dna phenotyping” | 0 | 0 |
| 8/14/2025 | European Journal of Human Genetics | <https://www.nature.com/ejhg/> | “facial phenotyping” | 14 | 0 |
| 8/14/2025 | European Journal of Human Genetics | <https://www.nature.com/ejhg/> | “facial” AND “DNA” AND “phenotyping” | 92 | 0 |
| 8/14/2025 | American Journal of Biological Anthropology | <https://onlinelibrary.wiley.com/journal/26927691> | “facial dna phenotyping” | 0 | 0 |
| 8/14/2025 | American Journal of Biological Anthropology | <https://onlinelibrary.wiley.com/journal/26927691> | “facial phenotyping” | 0 | 0 |
| 8/14/2025 | American Journal of Biological Anthropology | <https://onlinelibrary.wiley.com/journal/26927691> | “facial” AND “DNA” AND “phenotyping” | 9 | 0 |

In case these initial searches were too narrow, CMM also applied the following search string: [All: phenotyp*] AND [All: face]. While these searches returned a total of N=4469 results, none (n=0) passed the initial screening of titles to warrant a more detailed inspection as shown in Table 2 below.

Table 2. Additional unsuccessful searches of five journals

| **Date of Search** | **Journal** | **Website** | **Search String Used** | **No. of results** | **No. of potentially relevant results** |
| --- | --- | --- | --- | --- | --- |
| 8/20/2025 | American Journal of Human Genetics | <https://www.cell.com/ajhg/home> | [All: phenotyp*] AND [All: face] | 1643 | 0 |
| 8/20/2025 | Human Genetics and Genomics Advances | <https://www.cell.com/hgg-advances/home> | [All: phenotyp*] AND [All: face] | 105 | 0 |
| 8/20/2025 | Genetics in Medicine | <https://www.gimjournal.org/> | [All: phenotyp*] AND [All: face] | 1022 | 0 |
| 8/20/2025 | European Journal of Human Genetics | <https://www.nature.com/ejhg/> | [All: phenotyp*] AND [All: face] | 630 | 0 |
| 8/20/2025 | American Journal of Biological Anthropology | <https://onlinelibrary.wiley.com/journal/26927691> | [All: phenotyp*] AND [All: face] | 1069 | 0 |

While these searches were not a comprehensive, systematic search of all available scholarly literature for discourse on DNA-based facial phenotyping and did not include all potential useful search strings, it is reasonable to assume that these searches would have located any specific normative guidance by these prominent professional organizations for human geneticists directly relevant to this commentary. It is possible that practice guidance or recommendations have been published by individual scholars or professional organizations beyond those included in our searches.
